# Surgical Aspects of Typhoid and Paratyphoid Fevers

**Published:** 1919-12

**Authors:** 


**Surgical Aspects of Typhoid and Paratyphoid Fevers.** By A. E. Webb-Johnson, D.S.O., M.B., Ch.B., Temp. Col., A.M.S. Pp. 190. London: Oxford Medical Publications. 1919. Price 10s. 6d. net.

This interesting and pleasantly-written book is based on the author's Hunterian Lecture delivered in 1917, and deals with cases admitted to 14 Stationary Hospital during the first two years of the war. By way of introduction there is a short historical account of the evolution of our clinical and pathological knowledge of these diseases, embellished with reproductions of portraits of the noted men whose observations have gradually built up the structure of our present conceptions. Indeed, the author takes such a considerable interest in medical history that he cannot refrain from further decorating his work by portraits of Henry, Prince of Wales, the earliest English example of a death from typhoid, and of the statue of Jenner at Boulogne, as the "founder of scientific protective inoculation."

The main portion of the book is devoted to a succinct but exhaustive account of the complications of the enteric group of fevers (which, by the way, the author frequently calls the "Typhoid" group), with directions for the surgical treatment of such as are amenable to operative interference. The fact, however, which is most strongly brought out by these chapters is the comparative rarity of complications in protected individuals and the few instances in which surgical treatment is necessary or particularly successful. It is, in fact, a gratifying tribute from a distinguished surgeon to the success with which his medical and pathological colleagues have wrested from him the opportunities of utilising his skill and experience.

Of course, the one outstanding instance of a typhoid complication in which surgical intervention is the only hope is perforation of the intestine. Of this there were fourteen examples in 2,500 cases, and only three in inoculated patients. Six underwent operation with one recovery, the remaining five all died. It is remarkable that in 821 typhoid patients who had been inoculated the incidence of perforation was only 0.36 per cent., whereas in 297 not inoculated the percentage of perforation was 2.02. An interesting account is given of the relation of appendicitis to enteric fevers, and of the puzzling cases in which a generalised peritonitis occurs without perforation or other macroscopic evidence of how it arose.

The other suppurative complications of the enteric fevers are very fully described, and the general impression derived from the author's account of them is that visceral abscess formation is rare, often difficult to diagnose and locate, and usually occurs in patients in a profound toxæmic or pyæmic condition, whose recovery is very doubtful even after prompt and efficient surgical treatment.

The last chapter deals with "carriers," and is an excellent review of the present position of the subject, though the author's deductions are based mainly on his experience of acute clinical cases.

The book will be found useful by physicians as well as surgeons, as it necessarily deals mainly with points on the confines of medicine and surgery; and if the complications with which it deals become progressively rarer in practice, all the more does it behove the physician to know about them if he would avoid unpleasant surprises in the *post-mortem* room. We commend this book therefore to all practitioners, who will find in it a valuable store of information presented in a very attractive form.

